# A Laterally Acquired Galactose Oxidase-Like Gene Is Required for Aerial Development during Osmotic Stress in *Streptomyces coelicolor*

**DOI:** 10.1371/journal.pone.0054112

**Published:** 2013-01-11

**Authors:** Recep Liman, Paul D. Facey, Geertje van Keulen, Paul J. Dyson, Ricardo Del Sol

**Affiliations:** 1 Faculty of Science, Department of Genetics, Usak University, Usak, Turkey; 2 Institute of Life Science, College of Medicine, Swansea University, Singleton Park, Swansea, United Kingdom; University of Ottawa, Canada

## Abstract

Phylogenetic reconstruction revealed that most Actinobacterial orthologs of *S. coelicolor SCO2837*, encoding a metal-dependent galactose oxidase-like protein, are found within *Streptomyces* and were probably acquired by horizontal gene transfer from fungi. Disruption of *SCO2837* (*glxA)* caused a conditional *bld* phenotype that could not be reversed by extracellular complementation. Studies aimed at characterising the regulation of expression of *glxA* showed that it is not a target for other *bld* genes. We provide evidence that *glxA* is required for osmotic adaptation, although independently from the known osmotic stress response element SigB. *glxA* has been predicted to be part of an operon with the transcription unit comprising the upstream *cslA* gene and *glxA*. However, both phenotypic and expression studies indicate that it is also expressed from an independent promoter region internal to *cslA*. GlxA displays an *in situ* localisation pattern similar to that one observed for CslA at hyphal tips, but localisation of the former is independent of the latter. The functional role of GlxA in relation to CslA is discussed.

## Introduction

Galactose oxidase proteins have captivated biochemists due to the peculiar mechanism with which this family of proteins catalyze alcohol oxidation. The chemical reaction involves the oxidation of primary alcohols (including D-galactose and polysaccharides with D-galactose) into aldehydes, and the reaction is associated with the conversion of dioxygen to hydrogen peroxide. The metalloradical complex at the active site of these proteins provides its unique catalytic properties. Crystallographic and spectroscopic studies of the galactose oxidase active site [1] have previously shown that the active site that drives catalysis is composed of a cross-linked Tyrosine-Cysteine protein radical coordinated to a copper ion; with this complex acting as a two electron redox unit [2], [3].

From a protein motif perspective, the galactose oxidase active site adopts a β-propeller tertiary conformation; composed of ‘kelch’ motifs. This motif, initially discovered in *Drosophila* (within the Kelch ORF1 protein) [4], is now thought to be evolutionarily-widespread across all three Kingdoms [5], although its primary sequence appears poorly conserved. Structurally it can be described as a protein fold conformed by four-stranded β-sheets and, in the case of galactose oxidase, seven such ‘kelch’ motifs are arranged into a ‘propeller’ shape [6]. Similarly, the related family of glyoxal oxidase proteins shares many of the structural features of galactose oxidase, particularly the presence of ‘kelch’ motifs and the active site structure, although its catalytic activity concerns the oxidation of aldehydes into carboxylic acids and therefore is not necessarily functionally homologous to galactose oxidase.

Although initially identified in filamentous fungi, galactose oxidase genes have now been recognised in prokaryotes and plants. Biotechnological applications in areas like biosensors or biopolymer functionalisation have driven a multitude of studies aiming at the optimisation of recombinant expression and the engineering of the enzyme’s active site to modify substrate specificity [7]. While the functional role of glyoxal oxidases has been explored to a certain depth within the context of lignin degradation by fungi [8], [9], the physiological role of its relative galactose oxidase has been more elusive. The only published work assigning a functional role for a galactose oxidase-like concerns the *fbfB* gene from the myxobacterium *Stigmatella aurantiaca*. Mutation of this gene abolished morphological development, specifically fruiting body formation [10]. A more recent contribution addresses the biochemical properties of a galactose oxidase-like encoded by *Streptomyces coelicolor* (SCO2837). This revealed that the protein can process primary alcohols to aldehydes, and, despite possessing a signal peptide, the majority remains associated to the cell surface. The presence of a weakly conserved putative sortase signal was inferred as the reason for cell wall associated localisation [11].

In *S. coelicolor*, a putative galactose oxidase is seemingly encoded as part of a genetic locus comprising a glycosyl transferase encoding gene (*SCO2836*, *cslA*). The latter has been mutated and shown to be required for aerial development. Additional characterisation of CslA indicated it interacts with the morphogenetic protein DivIVA, which apparently contributes to CslA *in situ* localisation to hyphal tips. Furthermore, CslA is needed to synthesize a β(1–4) glucan at the tips, which the authors proposed plays a role in preserving tip integrity during cell wall remodelling associated to hyphal tip extension [12]. de Jong and colleagues expanded on this theme by analysing the contribution of CslA to the assembly of chaplin fibrils mediating surface attachment and ultimately the formation of the hydrophobic sheath leading to aerial erection in *S. coelicolor* [13].

The complexity of the chain of events leading to aerial development in *Streptomyces coelicolor* is highlighted by the numerous genetic loci so far identified as required for aerial hyphae formation and their further differentiation into spores. Genes that, when mutated, impair aerial hyphae formation are labelled ‘*bld’*, due to the distinctive appearance of the colony devoid of aerial growth. Numerous *bld* genes have been characterised, with an overrepresentation of loci encoding for transcription or regulatory elements, with very few encoding structural proteins (*bldK*, *ramS*, chaplin genes). In many cases the regulatory mutations that impair development also affect secondary metabolite production [14], [15]. The requirement for a functional CslA during aerial development indicates that its cognate galactose oxidase-like encoding gene may also play a role during differentiation.

To address this and contribute to the very limited knowledge concerning bacterial galactose oxidase-like genes, in this paper we present data describing the functional role of SCO2837, the only putative galactose oxidase encoded by *S. coelicolor*. We showed that this protein is required for aerial development and its expression is independent from known developmental or stress related regulatory genes. We also present strong evidence for a horizontal gene transfer event as the cause of its acquisition by the genus *Streptomyces*.

## Results

### Evidence for Horizontal Gene Transfer in the Acquisition of *SCO2837* Orthologs by Streptomycetes

**Figure 1:**
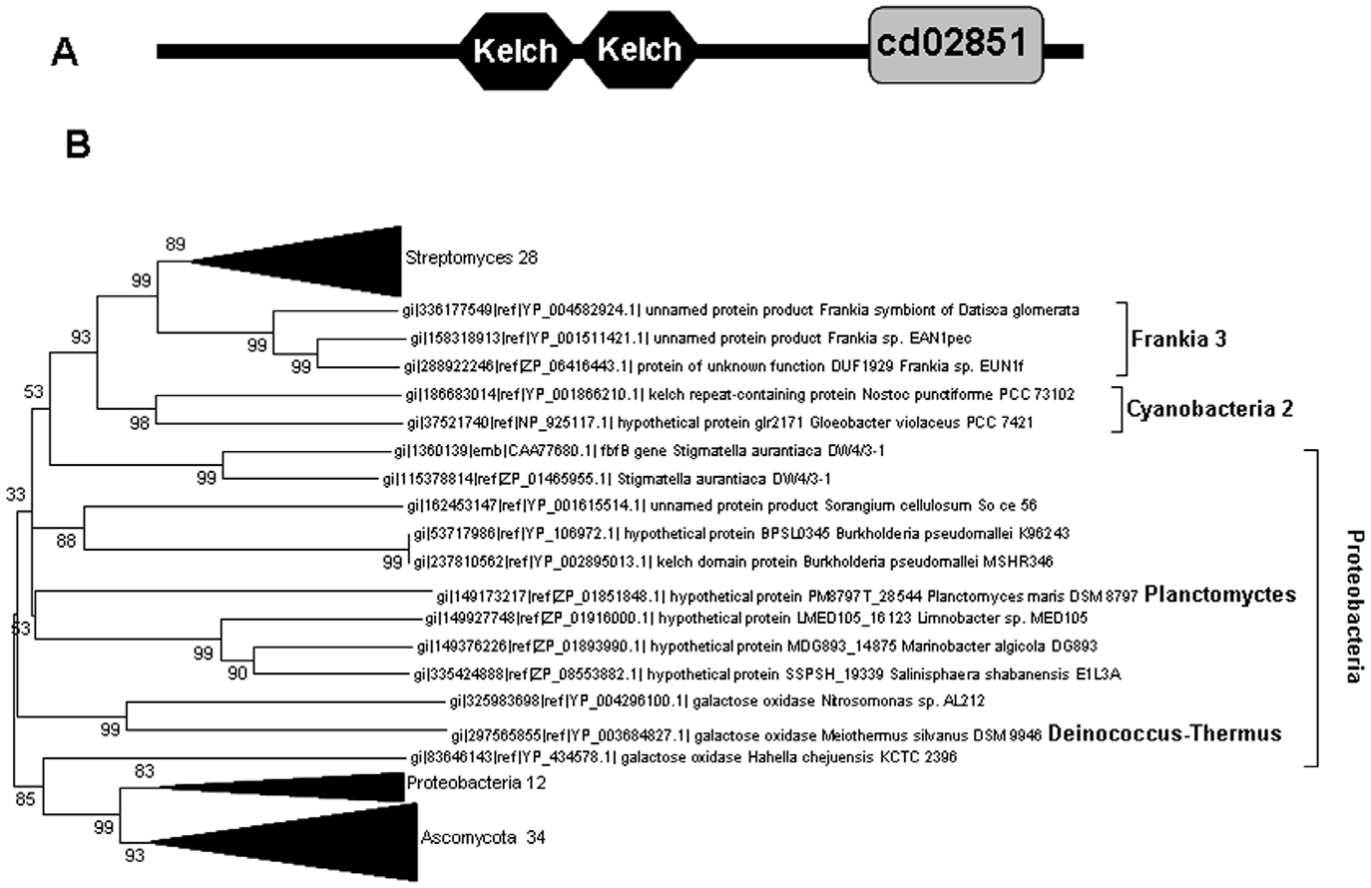
Phylogenetic analysis of SCO2837 (GlxA) orthologs. Sequences were selected based on protein domain organisation conservation as predicted by CDART (A). Neighbour-Joining Bootstrap phylogenetic tree generated using protein sequences displaying similar domain composition and organisation to GlxA (B). Clades conformed by sequences from a single phylum or class and displaying high bootstrap value are condensed, and the number of sequences within the clade indicated next to phylum/class name. Numbers at branch nodes indicate bootstrap values. A tree showing un-condensed clades is provided as Figure S2.

BLAST searches [16] within the *S. coelicolor* genome confirmed the presence of only one ORF (*SCO2837*) encoding for a putative galactose oxidase protein, as described [11]. Extended similar analyses to all sequenced *Streptomyces* genomes revealed a similar pattern, since all (28 genomes) but three encode for a single protein sharing SCO2837 protein domain architecture (Figure 1A). Tracking the evolutionary history of galactose oxidase-like proteins is a complex task due to the numerous protein sequences that despite containing the signature domain associated to this protein family (‘Kelch’ motif) are not members of this protein family. In order to compare true orthologs to SCO2837, a search based on conserved domain organisation was performed. After processing using the Conserved Domain Architecture Tool (CDART, NCBI), SCO2837 displays the typical domain composition found in galactose oxidase (GO) proteins (Figure 1A); namely a C-terminal E set domain (E_set_GO_C, cd02851) associated with the catalytic domain of galactose oxidase and two ‘kelch’ motifs (Kelch_3 Superfamily, cl02701). Although previously named *cslC* (cellulose synthase like, [12]), we have renamed *SCO2837* as *glxA* (galactose oxidase) and refer as such throughout this paper, as it is a more accurate depiction of the gene and its encoded protein.

The domain architecture prediction described above was used to retrieve all protein sequences displaying similar domain organisation and composition. The sequences thus obtained were aligned using ClustalW [17] and used to generate an un-rooted phylogenetic tree obtained using the Neighbor-joining method (MEGA5). Interestingly, most of the GlxA orthologs identified within Actinobacteria are found in *Streptomyces* (28), with only 3 belonging to other actinobacterial organisms (*Frankia*), while in other actinobacterial genera (e.g. *Mycobacterium)* orthologs are conspicuously absent. The remaining orthologs predominantly belonged to Ascomycota (34 sequences) and Proteobacteria (22 sequences), with one ortholog from Deinococcus-Thermus and Planctomycetes, respectively, and two from Cyanobacteria (Figure 1B). Reciprocal BLAST seraches, using homologous sequences from Ascomycota, resulted in the retrival of a similar group of *Streptomyces* sequences and confirmed the accuracy of our approach to select true orthologs.

Analysis of the phylogenetic distribution of GlxA orthologs supports the hypothesis that a horizontal gene transfer event has given rise to the presence of a coding sequence for this protein within *Streptomyces* genomes. Moreover, the almost ubiquitous nature of *glxA* orthologs in Streptomycete genomes suggests that this event occurred very early on in the radiation of *Streptomyces.* Except for three sequences in *Frankia*, the glaring absence of non-*Streptomyces* orthologs further supports the idea of acquisition by non-lineal descent. Interestingly, only three *Streptomyces* species (*S. roseosporus*, *S. albus* and *S. griseus*) encode more than one GlxA-like protein, suggestive of a more recent duplication within these genomes. In addition, the GC content of *glxA* (67%) in *S. coelicolor* and from other *Streptomyces*, even in those genomes where a duplicated locus exists, is significantly lower than the mean genome GC content (72.12%; *P*<0.05, Kruskall-Wallis). This GC content difference extends to the immediate upstream gene SCO2836 (*cslA*), encoding a protein displaying a glycosyl transferase domain. However, the GC content around this locus (20 Kb up- and downstream) is 72%, further reinforcing the notion that the *Streptomyces cslA-glxA* locus has been acquired by lateral gene transfer from an organism with a lower GC content genome. Similar comparisons in other *Streptomyces* genomes revealed that in all but one (*S. cattleya*) the sequence encoding the corresponding GlxA ortholog displays a lower GC content than the host genome (Table S1). Interestingly, the GC content of the *Stigmatella aurantiaca* galactose oxidase gene *fbfB* is significantly lower than that of the whole chromosome or adjacent genes. Just as we suggest for *Streptomyces*, the presence of a *glxA* ortholog within this organism may be the result of a horizontal gene transfer event.

Genome synteny analyses using assembled *Streptomyces* genomes revealed that *glxA* orthologs putatively form an operon with the upstream *cslA* gene, although it has never been experimentally confirmed. A third ORF is also conserved within this locus, downstream and converging on *glxA*, and encoding an endoglucanase. Interestingly, three genomes (*S. griseus*, *S. albus* and *S. roseosporus*) contain an extra copy of the *cslA*-*glxA* locus. One of these loci always displays synteny extending beyond these two genes with the other genomes while the other copy of the locus does not, which indicates that locus duplication events have occured within these organisms. In all *Streptomyces* genomes analysed this gene displays a GC content consistent with that of the overall chromosome. The *glxA* locus in *S. hygroscopicus* is devoid of the endoglucanase, and in some genomes it is not adjacent to *glxA* (Table S1). This genetic linkage between *cslA*, *glxA* and an endoglucanase encoding gene is only present in *Streptomyces*, as it is not conserved in other genomes encoding GlxA-like proteins. In *Stigmatella aurantiaca* the *glxA* ortholog *fbfB* (*STAUR*_4011) is located next to a gene encoding a glycosyl transferase protein, albeit divergently transcribed, and no endoglucanase encoding gene is present in the immediate genomic vicinity, as the nearest endoglucanase-like encoding gene is *STAUR*_4184.

### Disruption of *glxA* Impairs Aerial Development in a Medium-dependent Manner

The *SCO2837* (*glxA*) gene is part of conserved genetic locus with the up-stream gene *SCO2836* (*cslA*). The latter has already been characterised and shown to be required for aerial development in *S. coelicolor*, as well as contributing to the localisation of morphogenetic proteins [12], although such work did not address the potential contribution of *glxA* to morphological differentiation. In order to assign an *in vivo* function to *glxA* we disrupted this ORF using the widely used methodology based on *in vitro* transposon insertion mutagenesis [18], [19] and the Tn*5062*-mutagenised cosmid SCE20.2.H04. Since *cslA* and *glxA* display a putative operon organisation, it is possible that disruption of the former will result in polar effects on the latter. To determine if both genes were contributing equally to the same physiological events we also disrupted *cslA* using a similar methodology and Tn*5062*-mutagenised cosmid SCE20.2.D01. The allelic replacement in the insertion mutants obtained was verified by Southern blot (not shown).

**Figure 2:**
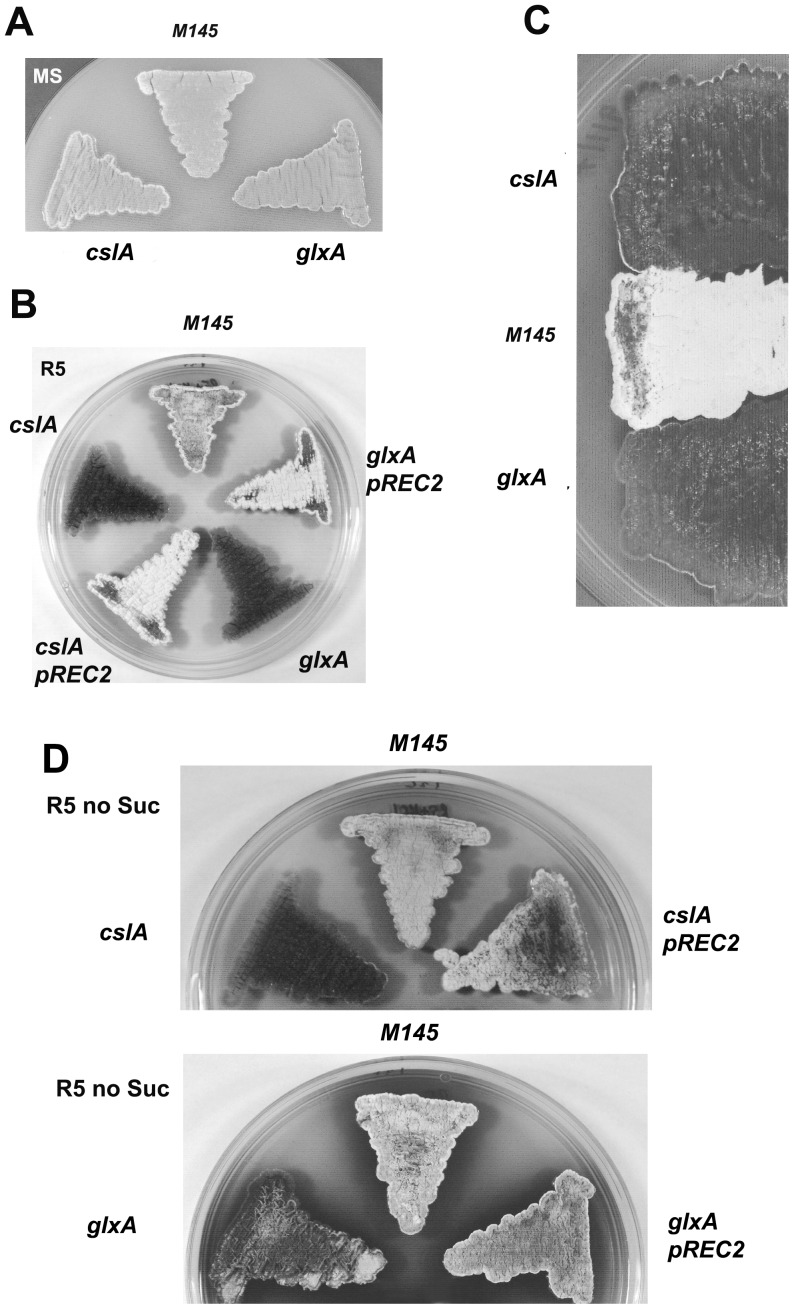
Phenotypic analysis of *glxA* mutant. *S. coelicolor* M145, *cslA^−^* (*cslA*-Tn) and *glxA* ^−^ strains are indistinguishable when grown on MS agar (A) but both mutant strains display a *bld* phenotype on R5 (B). The *bld* phenotype in mutants *cslA* ^−^ and *glxA* ^−^ cannot be rescued by extracellular complementation by the parental strain *S. coelicolor M145* (C). The *bld* phenotype of *glxA* ^−^ is conditional to the presence of high osmolyte in the medium. Strains shown were grown on R5 without Sucrose for 5 days (D).

Phenotypic analyses of the resulting mutants, DSCO2836 (*cslA*::Tn*5062*) and DSCO2837 (*glxA* ^−^), were performed by comparing the mutant strains to the parental *S. coelicolor* M145 strain when grown on different media. Growth on the surface of MS medium failed to reveal any phenotypic differences, as both parental and mutant strains showed a normal developmental progression in terms of aerial growth and pigmentation (Figure 2A). When grown on R5 medium, a clear distinction could be made between the parental *S. coelicolor* M145 strain and both *cslA*::Tn*5062* and *glxA* mutant strains. Here, in contrast to the parental M145 strain, both mutant strains were unable to erect aerial mycelia even after extended incubation, and therefore giving a clear *bld* phenotype (Figure 2B). Although this phenotype has been described for the *cslA* mutant, it is novel for *glxA* and in fact suggests that the *bld* phenotype associated with *cslA* may be partially contributed by a polar effect on its downstream gene, if they indeed are an operon. Both mutant strains were able to produce the pigmented antibiotics undecylprodigiosin and actinorhodin on R5 plates at apparently wild type levels. Examination of the *glxA* mutant using bright field and fluorescence microscopy failed to identify any relevant phenotypic traits as a consequence of the mutation. Hyphal morphology, branching and spore chain appearance (when grown on MS agar) resembled that of the parental M145 strain, setting apart the *glxA* mutant from a *cslA* mutant, where multiple sporogenic hyphae emerged from a common stalk [12].

In order to corroborate that the observed mutant phenotypes were linked to disruption of the respective genes, an integrative plasmid pREC2 was constructed to test for genetic complementation. This pSH152 derivative carries a 5.6 Kb insert containing both *SCO2836*-*SCO2837* genes and an extended upstream sequence to ensure the presence of the native promoter(s) driving their expression. Both mutant strains were successfully complemented with this plasmid (Figure 2B).

We further explored the *bld* phenotype displayed by both *cslA*::Tn*5062* and *glxA* ^−^ by attempting extracellular complementation. Both mutant strains were grown in close proximity to the parental M145 strain on the surface of R5 plates, but no apparent recovery of aerial development was observed after 5 days of incubation (Figure 2C). This observation places these novel *bld* genes outside the previously established *bld* hierarchy of extracellular complementation, reminiscent of what have been described for some other *bld* loci (*bldB* and *bldN*).

### Aerial Development of the *glxA* Mutant is Impaired by Hyper-osmotic Stress

We extended the exploration of the mutant phenotypes, focusing on the potential role of osmotic stress on the observed impairment of aerial development. Interestingly, when the *glxA* mutant was grown in R5 medium devoid of 10% sucrose, its ability to erect aerial hyphae was rescued. This phenotypic behaviour is limited to *glxA* ^−^, as the *cslA*::Tn*5062* mutant was unable to form aerial mycelium in both R5 and R5 lacking sucrose (Figure 2D). This phenotypic difference is extremely relevant as it provides evidence for a separate role for CslA and GlxA proteins during aerial development in the presence of high osmolyte, suggesting that the observed *bld* phenotype of the *cslA*::Tn*5062* mutant may not be attributed to a polar effect on *glxA* and challenging the notion of both genes been part of a polycistronic operon.

**Figure 3:**
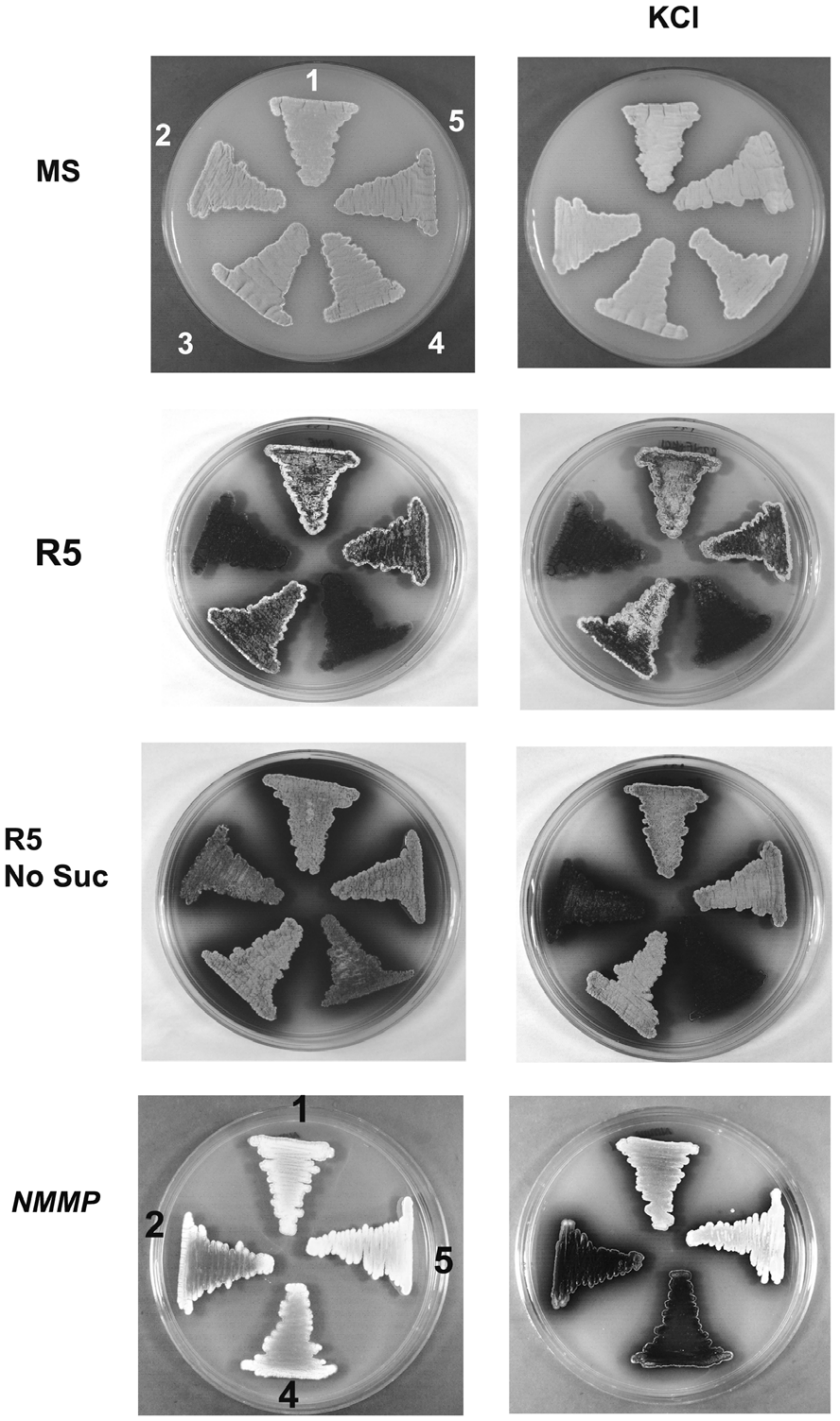
Aerial development of *glxA* ^−^ is impaired by hyper-osmotic stress in a medium dependent manner. All plates shown were incubated for 5 days. The plates shown on the right under the heading ‘KCl’ contain 250 mM KCl. The strains plated are: *S. coelicolor* M145 (1), *glxA* ^−^ (2), *glxA^−/^*pSH152 (4) and *glxA^−/^*pREC2 (3, 5; two independent clones).

The contribution of a high concentration of osmolyte to the *bld* phenotype of *glxA* ^−^ was further investigated, using KCl at a concentration of 250 mM. While on MS the high salt concentration had no phenotypic effects, the addition of salt to R5 lacking sucrose induced the *bld* phenotype. Since R5 is a very complex medium, we tested whether the observed high salt concentration effect could be reproduced on NMMP - a medium of much simpler composition. Indeed, normal aerial development was impaired when NMMP agar plates were supplemented with 250 mM KCl, while normal aerial development was observed without addition of osmolyte (Figure 3).

### Expression of *glxA* is Developmentally Controlled but Independent of Known Developmental or Osmotic Stress Response Elements

Having determined a connection between adaptation to osmotic stress and *glxA*, we set out to seek a link between its expression and known osmotic stress regulatory elements. The initial approach was to define a *glxA* expression pattern in relation to development. A transcriptional fusion was created, sub-cloning, in front of a *luxAB* promoter-less cassette (plasmid pRLux87), a 3487 bp DNA fragment encompassing the sequence upstream of the *SCO2836*/*SCO2837* putative operon and including the first 559 nt from *SCO2836* coding sequence. The resulting integrative plasmid pREC3 permits the monitoring of promoter activity within the cloned DNA fragment by means of detecting the luciferase activity encoded by *luxAB*.

**Figure 4:**
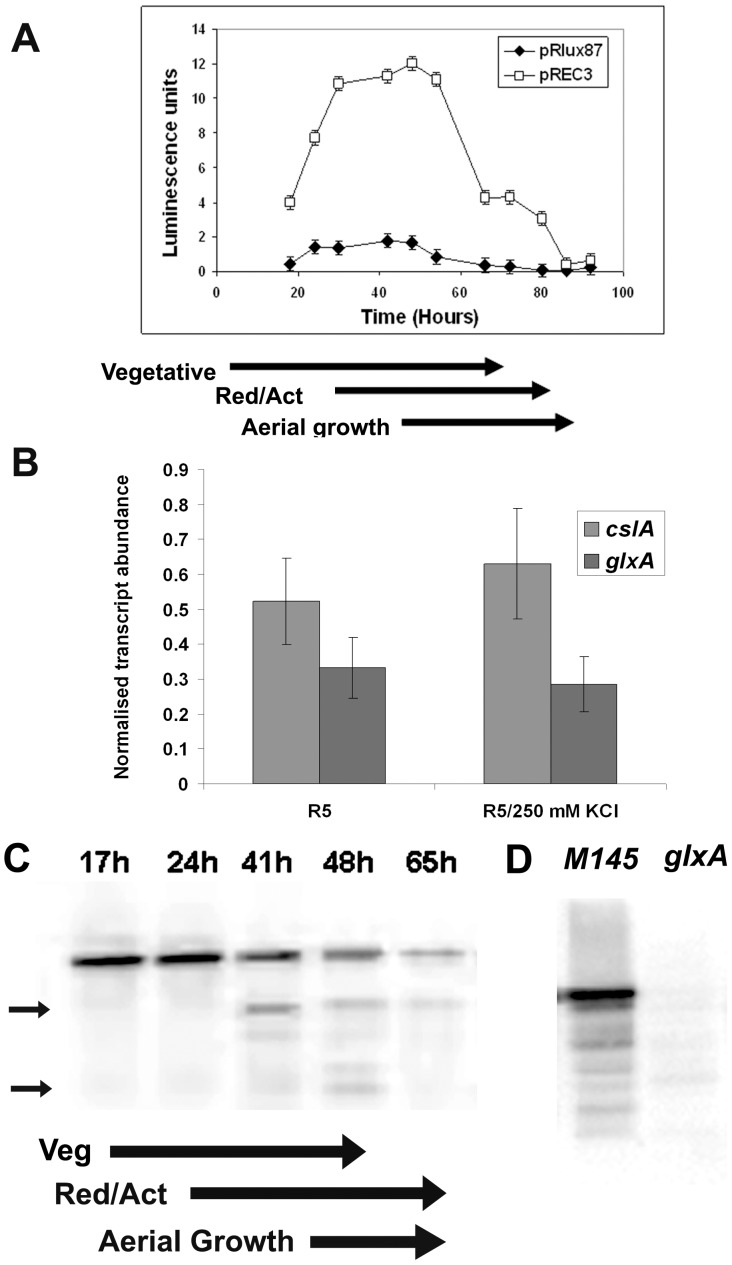
Expression of *cslA* and *glxA* is down-regulated at the onset of aerial development. The promoter activity of the sequence upstream of *SCO2836*/*SCO2837* was determined during growth on R5 medium, using a transcriptional fusion to *luxAB* (pREC3) in *S. coelicolor* M145. As negative control, the same strain carrying the vector pRLux87 was used. Onset of aerial development was estimated by visual inspection. Error bars indicate standard deviation (A). Quantification of *cslA* and *glxA* transcript abundance in R5 and R5 containing 250 mM KCl. Error bars indicate standard deviation (B). Imunodetection of GlxA in total protein extracts of *S. coelicolor* M145 grown on R5 plates during the times indicated (hours). Ten micrograms of total protein were loaded in each lane (C). Antibiotic production and aerial development were assessed by visual inspections prior to sample collection (Act/Red = Actinorhodin/Undecilprodigiosin, Veg = Vegetative). Small arrows indicate putative degradation products. No signal corersponding to GlxA was detected in a *glxA* mutant (D).

The pREC3 plasmid was introduced into *S. coelicolor* M145 and the resulting recombinant strain plated on the surface of R5 in black 96-well plates. In parallel, plasmid pRLux87 was also introduced into *S. coelicolor* M145 and used as negative control. Samples were processed as described in Experimental Procedures, while developmental stage and pigmented antibiotics production was monitored by visual inspection. As shown in Figure 4A, there is an obvious correlation between luciferase activity (and therefore promoter induction) and developmental stage. Promoter induction decreases from around 50 hours, concomitantly with the erection of aerial mycelium and sporogenesis. Although this is a gross estimation of promoter activity, it provides useful guidance in terms of linking the putative expression of the genes under study to relevant developmental phases. The observed lower expression levels associated with sporulation suggests that, once aerial development is triggered, *cslA* and *glxA* are downregulated, consistent with their role in early aerial hyphal erection. Quantification of *cslA* and *glxA* transcript abundance using qRT-PCR showed a typical expression pattern associated to operons, namely a decrease in transcript abundance in the second gene of the operon when compared to the first one (Figure 4B). The presence of high concentration of osmolyte (250 mM KCl) did not affect significantly the expression pattern of both genes.

Since the above promoter probe experiment is based on the assumption that *cslA* and *glxA* form a putative operon, we tried to confirm that *glxA* expression follows a similar pattern using an alternative approach. The *glxA* developmental control was confirmed by immunoblot, detecting GlxA protein abundance levels throughout development. *S. coelicolor* M145 was grown on the surface of R5 and total protein samples prepared from mycelium collected at various time points, determined by developmental stages (vegetative, early aerial, and sporulation). As observed in Figure 4C, GlxA protein levels were reduced in samples associated with late aerial hyphal development and/or sporulation, in agreement with the earlier observations using a promoter probe approach. Interestingly, we detected putative GlxA degradation products (Figure 4C, small arrows) in samples collected during aerial growth. This may suggests that a post-transaltional control mechanism is in place to modulate GlxA protein levels in aerial hyphae, in addition to downregulation of gene expression. No signal corresponding to GlxA or its degradation products was detected in samples obtained form *glxA* ^−^ (Figure 4D) grown for 2 days.

**Figure 5:**
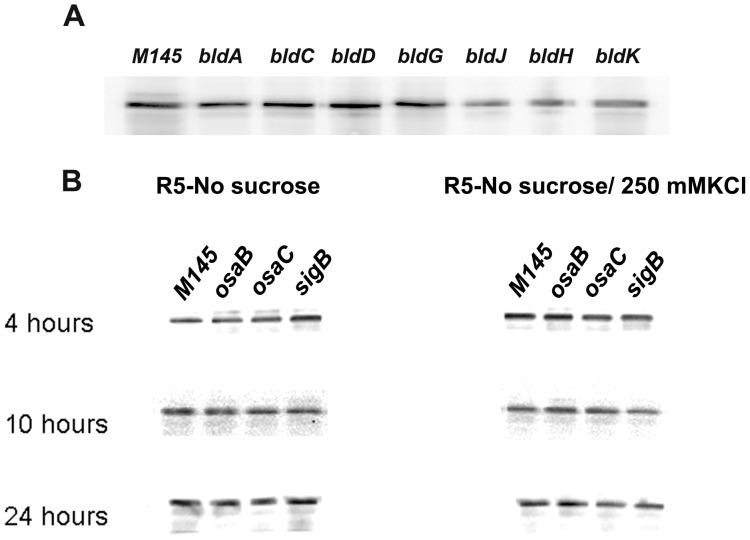
GlxA expression is not under the control of known developmental (A) or stress-response elements (B). Immunoblots showing GlxA protein abundance in various developmental mutants. Strains used (M145, *osaB*, *osaC* and *sigB*) are indicated on each gel lane. Ten micrograms of total protein were loaded in each lane.

We then proceeded to explore if GlxA protein expression levels are influenced by mutation of genes involved in developmental control. *bldA*, *bldC*, *bldD*, *bldG*, *bldJ*, *bldH* and *bldK* mutant strains were grown on the surface of R5 medium and protein samples prepared after 2 days. *S. coelicolor* M145 was used as positive control and 10 µg of total protein from each sample used for immunodetection of GlxA by Western blot. As shown in Figure 5A, not significant differences in protein abundance were observed. This result provides support to our earlier observations reporting that the *bld* phenotype associated with *glxA* lies outside the *bld* hierarchy. It also indicates that the contribution to morphological development by GlxA takes place independently and in parallel to the mechanisms used by other *bld* genes during aerial differentiation.

Since osmotic stress seems to play a relevant role in the *bld* phenotype associated with *glxA* disruption, we explored the potential dependence of GlxA protein expression levels on known osmotic stress response elements. Protein samples were collected from *sigB, osaB* and *osaC* mutant strains, using *S. coelicolor* M145 as control, after growth on R5 lacking sucrose and sucrose-less R5 supplemented with 250 mM KCl. In both conditions no differences in GlxA levels were detected, which indicates that its expression control is not directly induced by high osmolyte concentration or mediated by SigB, OsaB or OsaC (Figure 5B). We also used qRT-PCR to quantify the transcript abundance of both *cslA* and *glxA* in the absence and presence of high osmolyte. No significant induction of expression in response to osmotic stress was detected (not shown); suggesting that although functional GlxA is needed for aerial development under osmotic stress, its expression is not directly influenced by this environmental condition.

### *glxA* Expression is Driven by an Independent Promoter Situated within the Coding Sequence of its Upstream Gene

A *cslA* disruption mutant can be complemented by *cslA* on its own (Xu et al., 2008), which indicates that minor or none polar effects on *glxA* expression result from disrupting the upstream *cslA*. Our phenotypic characterisation of the respective mutants suggests that both CslA and GlxA contribute independently to aerial hyphal development, therefore *glxA* must possess its own promoter sequence; independent from a putative, yet to be identified, promoter upstream of *cslA*. To confirm this hypothesis we set out to clarify two fundamental questions. Firstly, whether the disruption of *cslA* prevents or affects *glxA* expression. Secondly, if we could gather evidence for a putative promoter region within the *cslA* coding sequence and driving *glxA* expression.

We decided to explore if expression of *glxA* in the *cslA* mutant background strain was unaffected. To correct for any putative read-through promoter activities from the transposon Tn*5062* disrupting the *cslA* gene and therefore artificially driving the expression of the downstream gene, we constructed a second Tn*5062*-derived disruption mutant using transposant cosmid SCE20.1.A11 (Figure S1). The resulting mutant strain DSCO2836b (*cslA*::Tn*5062b*) was phenotypically identical to the *cslA*::Tn*5062* mutant, but carries the transposon inserted in an opposite orientation compared to that in *cslA*::Tn*5062* and provides a useful control to determine if any expression of *glxA* is due to, or is independent of, read-through activities provided by Tn*5062*.

**Figure 6:**
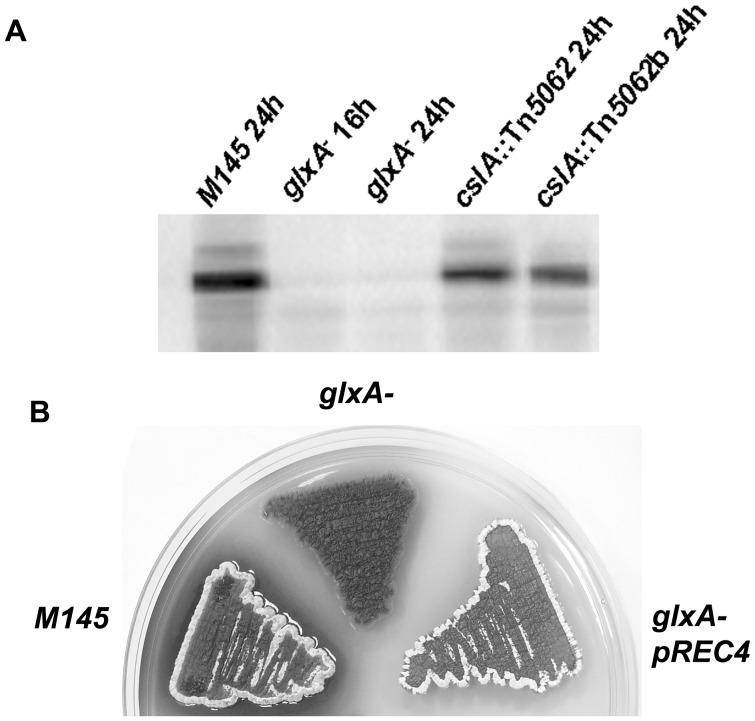
Only disruption of *glxA* abolishes production of GlxA. A- Parental and mutant strains were grown on R5 plates for the time indicated bellow and total protein samples prepared at the indicated times. Ten micrograms of total protein were loaded in each lane. Strains used were *S. coelicolor* M145 24 h, *glxA* ^−^ 16 h, *glxA* ^−^ 24 h, *cslA*::Tn*5062* 24 h and *cslA*::Tn*5062*b 24 h. B- A putative promoter internal to *cslA* supports expression of *glxA* and allows aerial development. Strains shown were grown on R5 plates for 4 days.

GlxA abundance was determined by immunoblotting protein samples obtained from *S. coelicolor* M145, *glxA* ^−^, *cslA*::Tn*5062* and *cslA*::Tn*5062b* grown on R5 plates. Total protein samples were collected during vegetative growth only, when GlxA expression is adundant. As expected, disruption of *glxA* results in no detectable protein (Figure 6A, lanes 2 and 3). Interestingly, disruption of *cslA* in both *cslA*::Tn*5062* and *cslA*::Tn*5062b* did not prevent production of GlxA (Figure 6A, lanes 4 and 5). The use of mutants carrying Tn*5062* inserted in both orientations indicates that the observed GlxA expression is unlikely to be due to read-through from a promoter within the transposon (transcriptional terminators prevent read-through from the apramycin resistance gene; [18], [19]), but more likely due to a promoter activity provided by promoter sequence(s) internal to the *cslA* coding sequence. The above observations also illustrate the advantages of using insertion mutants opposite to whole gene in-frame deletions. In this study the use of gene deletions would have resulted in overlooking and failing to detect the existence of an alternative promoter region within *cslA* and driving *glxA* expression.

Plasmid pREC4 was constructed to explore the existence of a promoter region located within the 3′ half of the *cslA* coding sequence, and driving expression of *glxA*. This construct includes *glxA* and 638 bp upstream of its start codon. This upstream sequence was deemed long enough to contain any putative promoter(s) controlling *glxA* expression. This integrative plasmid was introduced into *glxA* ^−^ and the resulting recombinant strain plated on R5 medium alongside the parental M145 strain and *glxA* ^−^ with the empty vector pSH152 (Hygromycin resistant derivative of pSET152). As shown in Figure 6B, the cloned insert in pREC4 is sufficient to induce aerial development to the *glxA* mutant, confirming that an active promoter sequence is contained within the 3′-end of *cslA* open reading frame. Interestingly this plasmid failed to rescue aerial development in the *cslA* mutant (results not shown); confirming that the *bld* phenotype observed in this mutant is not the result of a polar effect on the downstream *glxA* gene.

To support the above findings we used the comparative genomics database PromBase (http://nucleix.mbu.iisc.ernet.in/prombase/index.htm) to explore *in silico* the *clsA* sequence and identify putative *glxA* promoter sequence(s). This tool has been used to annotate putative promoter regions within 913 bacterial genomes [20], and its predictions are based in assessing the bendability, curvature and stability of DNA sequences. These properties directly correlate with the formation of an open complex between RNA Polymerase and the promoter sequence, leading to the separation of DNA strands around the −10 region [21]. Searches in Prombase pre-calculated data for *S. coelicolor* resulted in the identification of a sequence within the *clsA* open reading frame and predicted to be a promoter sequence with high probability. This putative promoter sequence spans from genomic position 3097115 to 3097192, around 270 nt upstream of *glxA* (position indicated in Figure S1) and contained in the insert carried by plasmid pREC4 which complements *glxA* ^−^.

### Sub-cellular Localisation of GlxA Reveals Association to the Cell Surface in a Tissue Specific Manner

**Figure 7:**
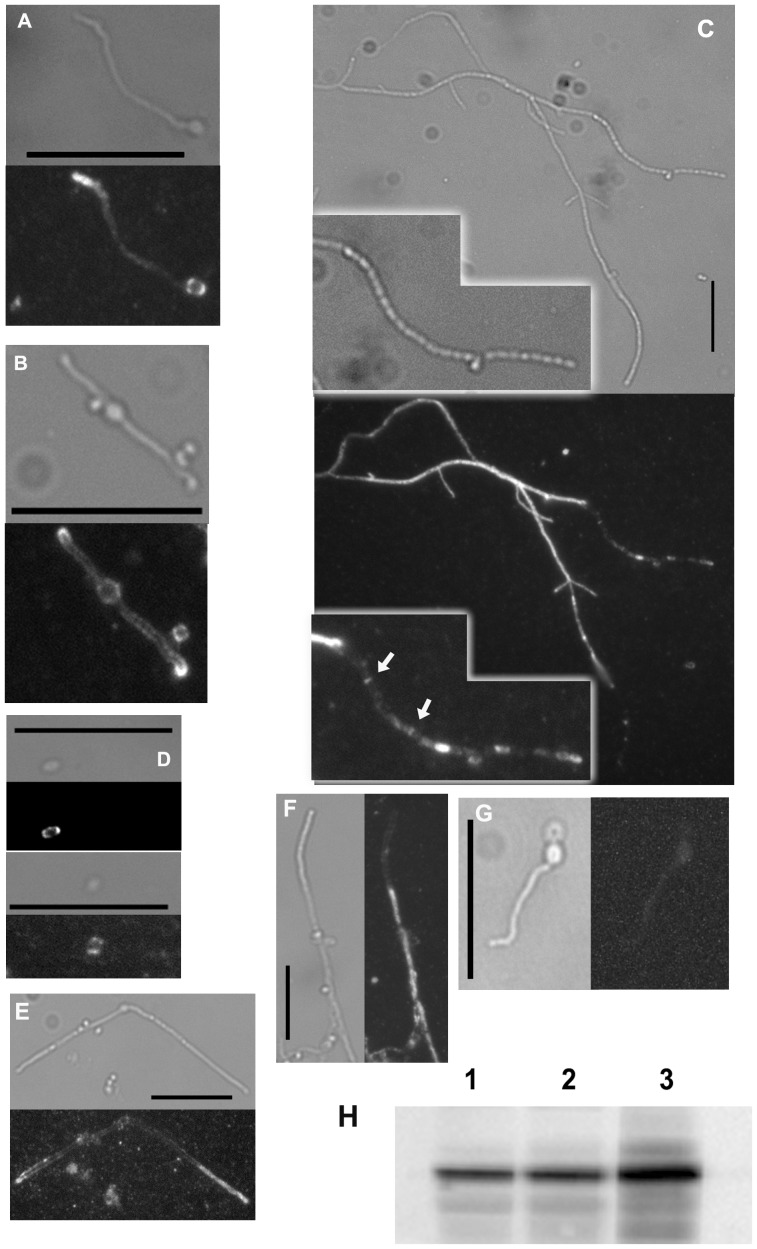
Subcellular localisation of GlxA. Immunomicroscopy showing preferential association of GlxA to growing hyphal tips (A, B) and sub-apical compartments in sporogenic hyphae (C). Bright light field and corresponding fluorescence field are shown. Insets in panel C highlights sporogenic hypha (brightfield) and the association of GlxA to sporulation septa (fluorescence). Panel D shows polar association of GlxA in detached spores. Panels E and F show *cslA* mutant displaying GlxA *in situ* localisation similar to that observed in the parental M145 strain. Panel G shows *glxA* mutant processed in similar manner (negative control). Bar: 10 µm. GlxA is not covalently associated to the cell surface (H). *S. coelicolor* M145 cells were grown on R5 for 24–30 h and total protein samples from sub-cellular compartments prepared. 1) Total protein, 2) Cell-membrane fraction, 3) Supernatant obtained from 1% SDS washing treatment of intact cells.

Previous work has shown that GlxA, despite possessing a putative signal peptide sequence, is predominantly associated to the cell envelope rather than secreted [11], [22]; but there have been no previous attempts to visualise its sub-cellular location *in vivo*. We explored the *in situ* localisation of GlxA in *S. coelicolor* M145 grown on the surface of R5 medium. As negative control we used the *glxA* mutant. Samples were collected after 24 hours and 48 hours incubation and subjected to immunomicroscopy using a rabbit anti-SCO2837p antibody and an anti-rabbit TexasRed conjugate. Since no fixatives were used, only target protein exposed on the cell surface was detected. The earlier time point allowed for visualisation of germinating spores while the extended incubation permitted visualisation of mature aerial hyphae bearing spore chains. The images obtained clearly show a localised abundance of GlxA at hyphal tips in growing germ tubes (Figure 7A, B). In older mycelium, the protein is mainly detected on the surface of non-sporogenic hyphae or the sub-apical portion of sporogenic ones, with only rare and weak fluorescent foci detected at the apical region of sporogenic hyphae (Figure 7C). In the latter, the protein seems preferentially associated to sporulation septa (Figure 7, C inset). Images from detached spores further reinforced this notion, as the fluorescent foci predominate at polar positions in the spores, presumably where the septa was formed earlier (Figure 7D). Similar experiments performed in the *cslA* mutant (Figure 7E, F) revealed a localisation pattern akin to that of the parental M145 strain, indicating that a functional CslA is not required for the localisation of GlxA. No fluorescence was detected in the negative control samples (Figure 7G).

In parallel, cell-free protein samples from different sub-cellular compartments were prepared. *S. coelicolor* M145 was grown on R5 and mycelium collected after 3 days. After sonication and low speed centrifugation to remove unbroken cells, the sample was subjected to ultracentrifugation to recover a cell-wall/cell-membrane fraction. At the same time, unbroken cells were gently washed with a 1% SDS solution to extract proteins non-covalently associated to the cell surface. Both cell-wall/membrane and cell surface associated samples were used for immunoblot to detect the presence of GlxA, alongside a total protein control sample. It was possible to detect the protein in both cell envelope and cell surface associated fractions (Figure 7H).

The above observations are in agreement with previously published data [11], [22], although the fact that GlxA can be recovered after a gentle SDS wash casts doubts on the possibility of the protein been covalently attached to the cell surface via sortase targeting as suggested [11].

## Discussion

### Streptomyces GlxA-like Coding Sequences were Laterally Acquired

The current wealth of data on structural and enzymatic properties of galactose oxidase (GO)-like proteins, mainly encoded by fungi, disguises the conspicuous absence of genetic and phenotypic observations derived from the analyses of mutants linking this group of proteins to physiological events. This highlights the need for such studies to be completed, in order to enhance our understanding of the *in vivo* role of these proteins. GO-like proteins of microbial origin have been particularly neglected, as only two studies attempting to assign a functional role to members of this protein family have been published. The *fbfB* gene from *Stigmatella aurantiaca* encodes a GO-like protein seemingly involved in morphological development [10]. In *Streptomyces coelicolor* a preliminary study on SCO2837 (GlxA) has been published, exhaustively exploring its enzymatic properties, but without adding a significant insight into its *in vivo* function [11].

We have taken advantage of the availability of completed genome sequences to produce a somehow comprehensive view of GO-like proteins from a phylogenetic perspective, aiming at elucidating their evolutionary origin in *Streptomyces*. The approach used to retrieve GO-like orthologs was based on protein domain composition conservation, in order to avoid the inclusion of false orthologs only sharing partial protein motif conservation. This approach retrieved sequences belonging to a narrow group of phyla, namely Ascomycota, Proteobacteria and Actinobacteria. The latter deserves special focus, as it is composed of mainly *Streptomyces* sequences, with only three orthologs from *Frankia* as non-*Streptomyces* representatives.

Since no archaeal orthologs were identified and only sequences of proteobacterial and actinobacterial origin were found within prokaryotes, it is reasonable to hypothesize that a GlxA-like protein was not encoded by the last common bacterial ancestor and probably arose during the evolution of the Eukarya lineage. Although, a widespread gene loss across all archaeal and most bacterial genomes could explain our findings, the most parsimonious interpretation is more likely. Thus, we suggest that *glxA*-like sequences arose within Eukaryotes and horizontal gene transfer events mediated their acquisition by the last common ancestor of a limited number of bacterial species. The significantly lower GC content of *glxA* orthologs in *Streptomyces* further reinforces the latter explanation. Additionally, the high boot-strap values associated to the Ascomycota and one of the Proteobacteria clades suggest that a similar event led to the acquisition of the *glxA*-like genes by Proteobacteria. Of particular note is the fact that *Stigmatella aurantiaca*’s *glxA* ortholog (*fbfB*) possess a GC content lower than its host chromosome, suggesting that a low GC content organism may have acted as donor in a gene transfer event, as we predict for *Streptomyces*.

### GlxA is Required for Aerial Development during Osmotic Stress in a Medium-dependent Manner

Phenotypic analyses of a *S. coelicolor glxA* mutant revealed a requirement for this gene during aerial development, albeit in a medium- dependent manner. Upon addition of high concentration of osmolyte to rich media, aerial development was impaired and led to a *bld* phenotype, although in the absence of the osmotic stress the normal morphological differentiation is restored. This phenotypic trait differentiates *glxA* from its cognate *cslA*, as mutation of the latter results in a *bld* phenotype that is not dependent on osmotic stress.

Despite the clear genetic linkage suggesting that *glxA* and *cslA* contribute to the same metabolic pathway leading to aerial development, their contributions are not synonymous in certain environmental conditions like osmotic stress. Although the *in vivo* substrates and the biochemical reactions performed by CslA and GlxA in *Streptomyces* remain to be confirmed by experimentation, it has been proposed that CslA contributes to the synthesis and extrusion of cellulose fibrils, which serve as an ‘anchoring point’ for chaplins fibrils required for their surface attachment [13]. Alternatively, Xu and colleagues demonstrated that CslA plays a role in hyphal tip growth, apparently via the synthesis of a β(1–4) glucan during cell wall remodelling at the hyphal tip [11].

Incorporating GlxA into this sequence of events is more challenging, since its substrate molecule remains to be identified. Our findings confirm that GlxA is abundantly expressed during the transition phase leading towards aerial development, and then down-regulated. The *in situ* visualisation of GlxA revealed preferential hyphal tip association not dependent on CslA. This result indicates that both GlxA and CslA may contribute to the localised cell wall remodelling associated with tip growth. Galactose oxidase converts alcohols into aldehydes and reduces dioxygen to hydrogen peroxide. The signalling role of hydrogen peroxide is well documented in plants [23] and GlxA may play a similar role in *Streptomyces*, namely the generation of hydrogen peroxide which acts as a signalling molecule [11]. However, this is an unlikely proposition as we failed to rescue aerial deveopment in a *glxA* mutant by adding hydrogen peroxide in various doses and formats (not shown).

The *glxA* genetic locus offers clues for an alternative explanation for its functional role. We propose that, during hyphal tip remodelling, the β(1–4) glucan synthesised by CslA is the subject of hydrolysis by an endoglucanase, and products of this reaction may feed enzymatic pathways mediated by exoglucanase(s) that in turn generate a primary alcohol containing substrate molecule for GlxA to process. The endoglucanase gene (*SCO2838*) genetically linked to the *cslA* and *glxA* locus could contribute to this pathway. The three enzymes could act in a concerted manner, where CslA synthesises a localised polysaccharide needed for cell wall remodelling at the hyphal tip, while the endoglucanase ensures its localised turnover, and a by-product from this reaction serves as substrate for GlxA. In a *glxA* mutant, the accumulation of its unprocessed substrate would cause an inhibition of the endoglucanse activity, which in turn would prevent further synthesis by CslA and correct cell wall remodelling. The gene upstream of *cslA* encodes a peptidoglycan binding protein, further supporting a ‘cell wall remodelling role’ for this genetic locus in *Streptomyces*.

During hyperosmotic stress the cell wall probably undergoes additional and localised modifications to cope temporarily with the increased osmotic pressure, therefore requiring an even better orchestrated sequence of reactions involving CslA and GlxA. Under no osmotic stress the contribution of GlxA to the events proposed above is negligible so that a *glxA* mutant develops normally. Only when enhanced cell wall remodelling is required, as during osmotic stress, is the contribution of GlxA significant enough to be indispensable for correct aerial hyphae differentiation.

### *glxA* Expression is Driven by a Promoter Localised Internally to *cslA*

Despite the apparent bicistronic operon organisation of *cslA* and *glxA*, disruption of the former did not prevent the expression of the downstream *glxA*. We confirmed experimentally that there is a promoter region within *cslA* coding sequence and driving *glxA* expression to the necessary levels to provide genetic complementation of a *glxA* mutant. Although this result does not preclude the possibility of a polycistronic transcript encompassing both *cslA* and *glxA*, it certainly indicates the existence of an alternative regulatory mechanism modulating *glxA* expression independently from *cslA*. Furthermore, it casts some doubts on a putative operon organisation predicted by other authors [11], [12] and yet to be confirmed experimentally. Although genetically linked, CslA and GlxA act maybe cooperatively but certainly independently to ensure normal aerial development in *Streptomyces*. This is particularly relevant within the context of existing *bld* genes and the so called ‘*bld* hierarchical cascade’. The *glxA* mutant can not be complemented extracellularly by its parental wild type strain. This result is somehow unexpected, as GlxA is predicted to possess a signal peptide and therefore be secreted [11]. This putative secretion should provide sufficient ‘diffusible’ activity to induce extracellular complementation by a wild type strain in a *glxA* mutant. Our results indicate that GlxA remains attached to the cell surface, therefore explaining the lack of extracellular complementation observed.

In summary, we provide compelling evidence describing a new *bld* locus in *S*. *coelicolor* that was acquired by horizontal gene transfer from a lower GC content donor organism. We also propose a mechanism explaining the functional role of GlxA, incorporating existing evidence related to putative functional partners. The fact that the other GO-like functionally characterised in bacteria (*fbfB*) also plays a role in morphological differentiation in *S. aurantiaca* suggests a conservation of the functional role for this group of proteins, and reinforces the hypothesis of a common evolutionary source. Glyoxal oxidases are closely related in structure and function to GO, and therefore may share a common ancestor with GOs. In the phytopathogenic fungi *Ustilago maydis* a glyoxal oxidase encoding gene is essential for filamentous growth [24], which provides support for the idea of a common ancestor for these protein families, contributing to the correct balance of cell wall remodelling events and playing a central role in morphological differentiation.

## Experimental Procedures

### Bacterial Strains and Media

*Streptomyces coelicolor* A3(2) and *E. coli* strains are listed and described in Table 1. Cloning procedures were performed in *E. coli* JM109, while *E. coli* ET12567/pUZ8002 was used for intergeneric conjugative transfer of plasmid DNA into *Streptomyces* strains [25]. Microbial cultures were performed as described for *E. coli* strains [24]. *S. coelicolor* strains were grown on MS (mannitol soya flour) and R5 agar plates [26]. *Streptomyces* mutant strains were obtained using *Tn*5062-mutagenised cosmids (Table 1, [18]. The identity of all mutants was confirmed by Southern blot [26].

**Table 1:** Strains and plasmids.

Strain	Description	Transposon insertion^a^ (genome position)	Source
*S. coelicolor* A3(2) M145	Prototrophic SCP1– SCP2– Pgl+		[25]
DSCO2836, *cslA*::Tn*5062*	*M145 sco2836*::Tn*5062*	SCE20.2.D01 (3096287)	This study
DSCO2836b, *cslA*::Tn*5062*b	*M145 sco2836*::Tn*5062*	SCE20.1.A11 (3096499)	This study
DSCO2837, *glxA* ^−^	*M145 sco2837*::Tn*5062*	SCE20.2.H04 (3097685)	This study
*bldA*	*bldA39 hisA1 uraA1 strA1 SCP1* ^−^ *_ SCP2* ^−^		[33]
*bldC*	bldC18 mthB2 cysD18 agaA7 SCP1^NF^ SCP2*		[34]
*bldD*	bldD53 cysA15 pheA1 mthB2 strA1 SCP1^NF^ SCP2*		[34]
*bldG*	*bldG103 hisA1 uraA1 strA1 Pgl* ^−^ *_SCP1* ^−^ *SCP2* ^−^		[35]
*bldJ*	bldJ261 hisA1 uraA1 strA1 Pgl_ SCP1^NF^ SCP2*		[36]
*bldH*	*bldH109 hisA1 uraA1 strA1 Pgl* ^−^ *_SCP1* ^−^ *SCP2* ^−^		[35]
*bldK*	*bldK::aadA derivative of M145*		[37]
DSCO0600 *sigB* ^−^	*M145 sigB*::Tn*5062*	SC5G5.1.C05 (639940)	[38]
DSCO5749 *osaB* ^−^	*M145 osaB*::Tn5062	SC7C7.1.G11 (6285443)	[38]
DSCO5747 *osaC* ^−^	*M145 osaC*::Tn5062	SC7C7.1.D06 (6278735)	[38]
*E. coli* JM109	F’ *traD36 proA^+^B^+^ lacIq* Δ*(lacZ)M15/*Δ*(lac-proAB) glnV44 e14- gyrA96 recA1 relA1endA1 thi hsdR17*		[39]
*E. coli* ET12567 (pUZ8002)	*Dam13*::Tn*9 dcm6 hsdM hsdR recF143* 16z*jj201* ::Tn*10 galK2 galT22 ara14 lacY1* *xyl5 leuB6 thi1 tonA31 rpsL136 hisG4 tsx78 mtli glnV44*, containing the nontransmissible *oriT* mobilizing plasmid, pUZ8002		[40]
**Plasmids**			
pIJ2925	Ampicillin resistance		[41]
pSH152	Hygromycin resistance		[42]
pRLux87	Promoter-less *Vibrio harveyi luxAB* operon, *hyg^R^.* Derived from pRLux86.		[31]
pREC1	*sco2836*,*sco2837* in pIJ2925		This study
pREC2	*sco2836*,*sco2837 in* pSH152		This study
pREC3	promoter probe vector		This study
pREC4	Hygromycin resistance		This study

### DNA Manipulation and Plasmid Construction

DNA manipulation was performed using standard procedures [25]. DNA fragments used to construct plasmids are shown in Figure S1. Briefly, a 7397 bp XbaI fragment from transposon mutagenised cosmid SCE20.1.D.02 (http://strepdb.streptomyces.org.uk/) was sub-cloned into pIJ2925 to generate pREC1. The latter was digested XbaI/EcoICRI (5656 bp insert) and ligated to integrative plasmid pSH152 digested XbaI/EcoRV, resulting in pREC2. Promoter probe vector pRLux87 was digested BglII, then ligated to a 3487 bp BglII/BamHI fragment from pREC1 encompassing from the upstream sequence of *SCO2836* and up to the first 559 nt of *SCO2836* coding sequence. The plasmid obtained (pREC3) carries the promoter region of the *SCO2836*-*37* putative operon, upstream and driving the transcription of the promoter-less *luxAB* cassette. The direction of the cloned fragment was verified by restriction and sequencing.

Plasmid pREC4 was constructed as follows. pREC1 was digested BamHI to excise a 3.2 KB fragment containing *SCO2837* and 638 bp upstream sequence, covering the 5′-end of *SCO2837* ORF. The insert was sub-cloned into pSET152 digested BamHI, and the Apramycin resistant gene in this plasmid was replaced for Hygromycin using the PCR-targeted system [27].

### Sequence Data Analyses

Protein sequences were retrieved from NCBI (http://www.ncbi.nlm.nih.gov) and Broad Institute (http://www.broadinstitute.org). The Conserved Domain Architecture Tool ([28]; CDART, NCBI,) was used to retrieve protein sequences sharing SCO2837 domain organisation. The Phylogenetic reconstruction was performed in MEGA5 (Molecular Evolutionary Genetic Analysis, ver. 5.05, [29]) using the Neighbor-Joining method. The complete deletion option was selected to remove all missing data or alignment gaps. Bootstrap values (1000 replicates) were used to assess the robustness of the inferred phylogeny. Synteny analyses were performed using SynMap ([30]; http://genomevolution.org/CoGe/SynMap.pl). GC content was calculated using Gene Runner software (http://www.generunner.net/). Prombase (http://nucleix.mbu.iisc.ernet.in/prombase/) was used to identify putative promoter sequences upstream of SCO2837 start codon [20],[21].

To provide evidence that orthologs of *SCO2836* (*glxA*) and *SCO2837* (*cslA*) were extraneous in Streptomycete genomes, we compared the percentage GC content of each ortholog with, (i) the average percentage GC content of the host genome and, (ii) with the percentage of immediately adjacent genes. Nucleotide sequences for all genes were retrieved for completely assembled *Streptomyces* genomes. Pair-wise comparisons of gene versus genome percentage GC content were performed using Mann-Whitney tests (SPSS 13.9 for Windows).

### Immunodetection of Proteins

Total protein extracts were obtained from mycelium grown on the surface of cellophane discs placed on top of agar plates. After an overnight incubation (16 hours) the discs were transferred to plates containing the chosen stimulus, keeping an untreated sample as reference. Cells were scraped from the cellophane and suspended in Sonication Buffer (50 mM Tris-HCl, pH 8, 200 mM NaCl, 15 mM EDTA, Complete protease inhibitor cocktail [Roche Diagnostics]). Cells were disrupted by sonication (20 s burst on ice) and centrifuged (13 000 r.p.m. for 3 min) to remove unbroken cells. Total protein concentration was determined using the Bradford method (Bio-Rad). SDS-PAGE was performed as previously described [26]; loading 10 µg of total protein per lane in 10% SDS/PAGE gels. Proteins were transferred to PVDF (BioRad) by semidry electrotransfer ((Trans-Blot SD, Bio-Rad). Immunological detection was performed using an ECL Advance Western blotting detection kit (GE Healthcare). SCO2837 was detected using a rabbit anti-SCO2837 antibody diluted 1∶50000 (kindly provided by J. Whitaker; [11]), and a goat anti-rabbit HRP-conjugate (Sigma).

Sub-cellular compartment association samples were obtained as described [31]. Briefly, cells grown on cellophane discs on top of agar media were collected and sonicated as described earlier. A low speed centrifugation step removed unbroken cells. A total protein sample was collected at this stage, and the remaining sample subjected to centrifugation at 200,000×g for 1 h at 4°C to separate cell membrane fraction (pellet) from soluble fraction (supernatant). Soluble protein concentrations were determined using the Bradford method (Bio-Rad), and protein concentration in the total membrane protein fraction was determined using a PlusOne 2-D Quant kit (Amersham Pharmacia Biotech) after solubilization of the membrane pellet in Sonication buffer containing 50 mM 3-[(3-cholamidopropyl)-dimethylammonio]-1-propanesulfonate. For SDS-washing treatment, mycelium grown as above was gently washed in 1% SDS for a few minutes and then subjected to centrifugation. The supernatant was used to detect the presence of SCO2837p protein by Western blot.

### Gene Expression Detection and Quantification

Time course monitoring of gene expression was conducted using a transcriptional fusion to *luxAB* reporter cassette. Briefly, 2 µl from spore suspensions of equal concentration from strains under study were spotted on black 96-well microplates containing agar media of choice in each well. After suitable incubation at 30°C the plates were exposed to vapours of N-decanal (Sigma) for 3 minutes, and light emission was quantified with an Anthos Lucy 1 microplate luminometer, with 0.1 s integration. At least three independently grown cultures were assayed in this way, and a negative control, consisting of the strain under study carrying pRLux87, a plasmid containing a promoterless *luxAB* operon, was used in triplicate as well.

Transcript abundance was quantified by qRT-PCR as described [32]. Gene specific primer sets are listed in Table 2. Total RNA was extracted from three independent cultures (biological replicates) grown on R5/250 mM KCl plates; and each was then subjected to qRT-PCR in triplicate (technical replicates). *S*. *coelicolor hrdB* was used as an endogenous control to normalise samples.

**Table 2:** Oligonucleotides.

Oligonucleotides	Sequence (5′-3′)
SCO2836RTF1	AGACGATCCTCAAGCAGTA
SCO2836RTR1	ATCATCATCGTGTAGTTGAAGAG
2837RTF1	CGGCAACAACCAGGACAACTC
2837RTR1	GCGTCGGCACCTTCTTGATG
hrdBFor	CCTCCGCCTGGTGGTCTC
hrdBRev	CTTGTAGCCCTTGGTGTAGTC

### Immunomicroscopy

Spores from the *Streptomyces* strains under study were inoculated in the acute angle of coverslips inserted in the agar medium of choice and incubated for 3–4 days. The coverslips were removed and subjected to immunomicroscopy as described [25]. Cells were treated with rabbit anti-SCO2837 diluted 1∶2000 in 2% BSA/PBS and incubated overnight at 4°C. After a gentle wash with PBS, coverslips were treated with an anti-rabbit TexasRed conjugate (Invitrogen) diluted 1∶1000 in 2%BSA/PBS for 1 hour. Coverslips were gently washed with PBS, mounted on slides in a drop of 10% Glycerol solution. Images were obtained using a Nikon Eclipse E600 epifluorescence microscope fitted with a Coolsnap microscope camera (RS Photometrics., Tucson, AZ).

## Supporting Information

Figure S1 Diagram representing *SCO2837 (glxA)* genetic locus and DNA fragments used in sub-cloning experiments. The position of transposon insertions is indicated by gray vertical arrows, while the putative promoter predicted by PromBase is indicated by a striped arrow with chromosome position indicated. Refer to [19] for a detailed map of Tn*5062*.
(TIF)

Figure S2 Neighbour-Joining Bootstrap phylogenetic tree generated using protein sequences displaying similar domain composition and organisation to GlxA. Numbers at branch nodes indicate bootstrap values.
(TIF)

Table S1 GC content (%) of *Streptomyces* and *S. aurantiaca* genomes and *SCO2837* (*glxA*) orthologs used in GC content comparisons. Only assembled genome sequences were included. ORF name is indicated when more than one *glxA*-like sequence per genome is present.
(DOC)
