# Supplementary figures and images for: A Laterally Acquired Galactose Oxidase-Like Gene Is Required for Aerial Development during Osmotic Stress in Streptomyces coelicolor
Source: PLoS One. 2013 Jan 11;8(1):e54112. doi: 10.1371/journal.pone.0054112 (PMC3543389; doi:10.1371/journal.pone.0054112)

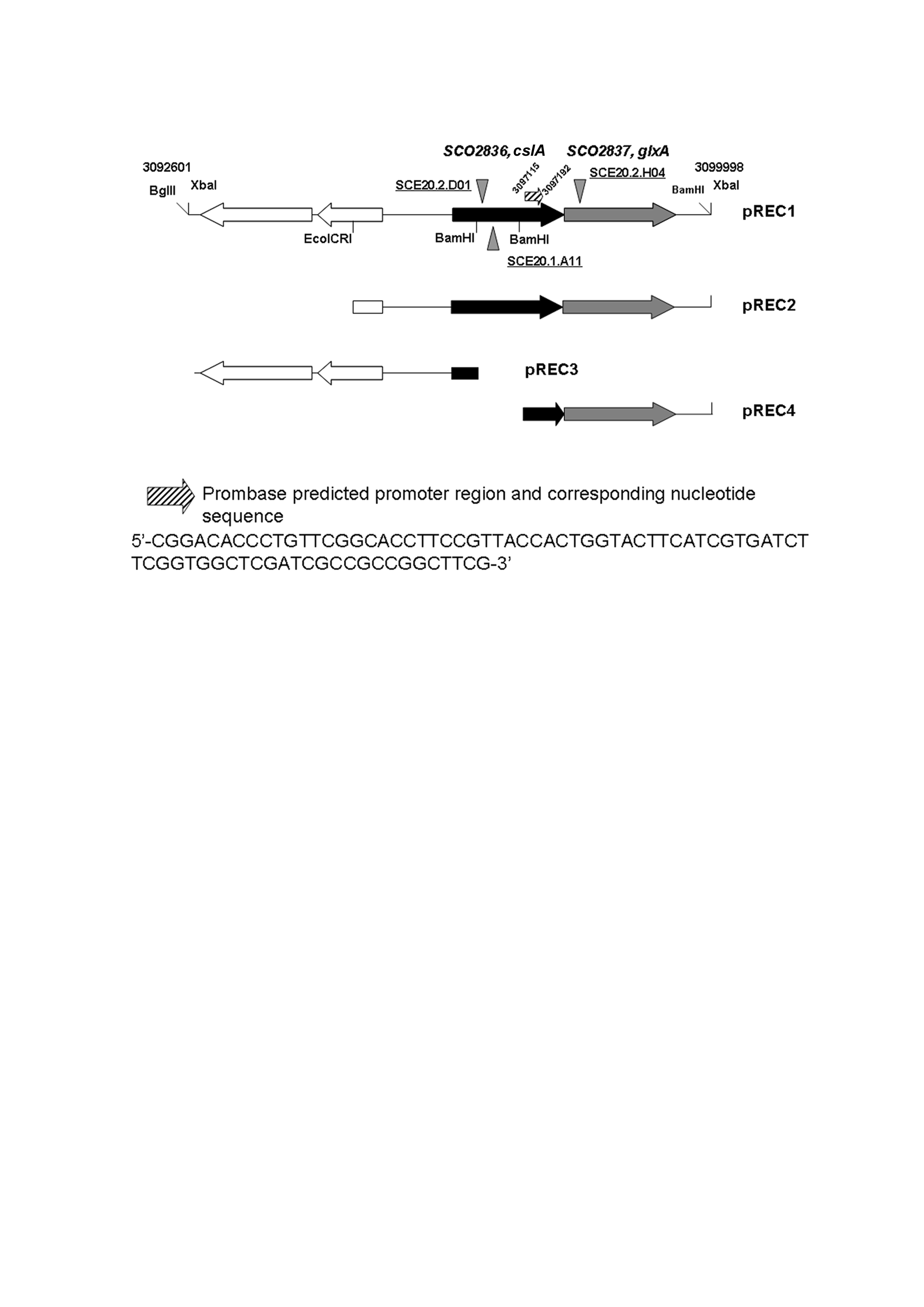

Supplement: Figure S1 — Diagram representing SCO2837 (glxA) genetic locus and DNA fragments used in sub-cloning experiments. The position of transposon insertions is indicated by gray vertical arrows, while the putative promoter predicted by PromBase is indicated by a striped arrow with chromosome position indicated. Refer to [19] for a detailed map of Tn5062. (TIF) [file pone.0054112.s001.tif]

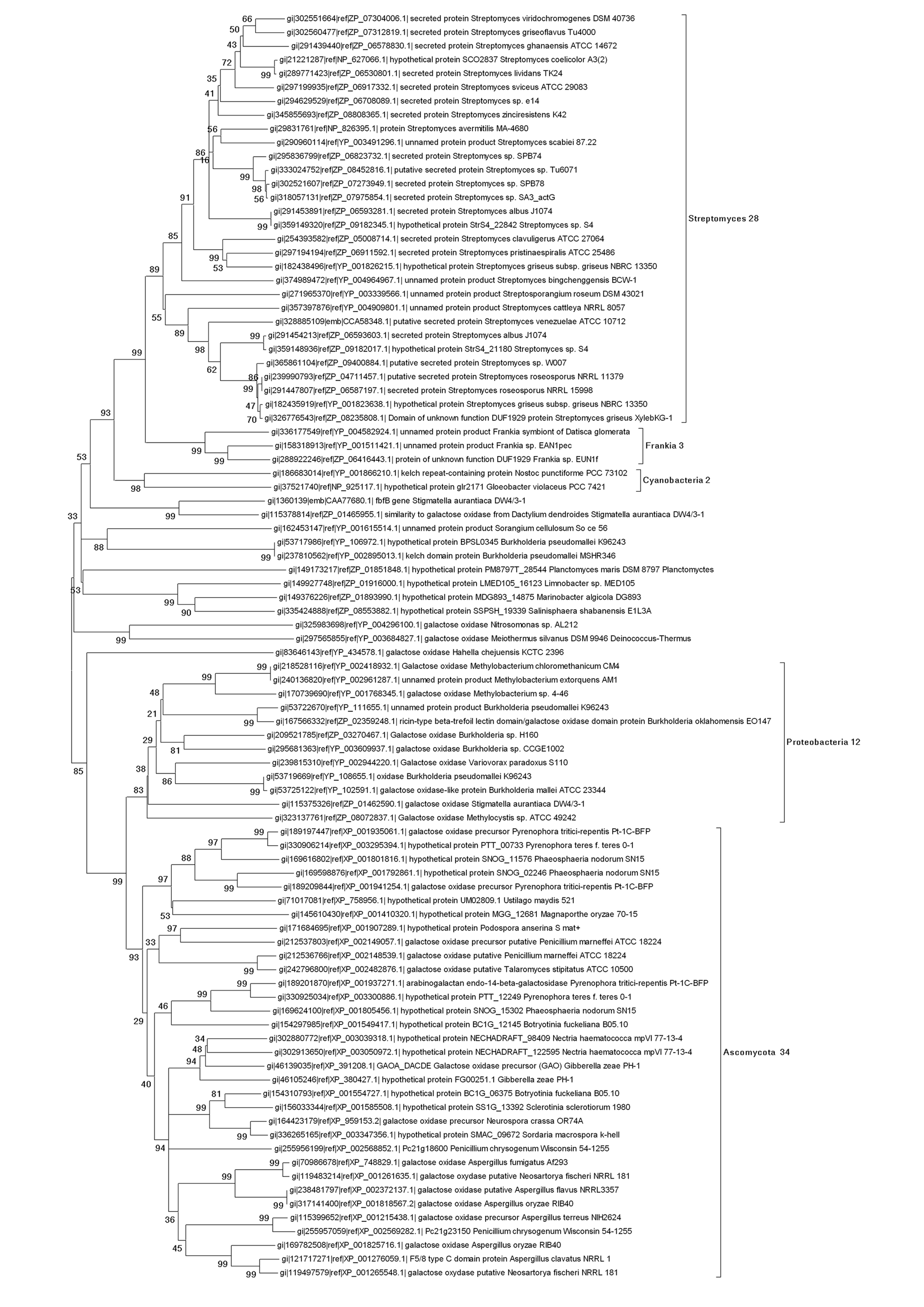

Supplement: Figure S2 — Neighbour-Joining Bootstrap phylogenetic tree generated using protein sequences displaying similar domain composition and organisation to GlxA. Numbers at branch nodes indicate bootstrap values. (TIF) [file pone.0054112.s002.tif]
