# Supplementary material for: A Laterally Acquired Galactose Oxidase-Like Gene Is Required for Aerial Development during Osmotic Stress in Streptomyces coelicolor
Source: PLoS One. 2013 Jan 11;8(1):e54112. doi: 10.1371/journal.pone.0054112 (PMC3543389; doi:10.1371/journal.pone.0054112)
Supplement: Table S1 — GC content (%) of Streptomyces and S. aurantiaca genomes and SCO2837 (glxA) orthologs used in GC content comparisons. Only assembled genome sequences were included. ORF name is indicated when more than one glxA-like sequence per genome is present. (DOC) [file pone.0054112.s003.doc]

**Supplementary Table 1:** GC content (%) of *Streptomyces* and *S. aurantiaca* genomes and *SCO2837* (*glxA*) orthologs used in GC content comparisons. Only assembled genome sequences were included. ORF name is indicated when more than one *glxA*-like sequence per genome is present.

| **Organism** | **Genome GC%** | ***SCO2837*-like GC%**  **(ORF name)** |
| --- | --- | --- |
| *S. coelicolor* A3(2) | 72.12 | 67.0 |
| *S. sp. SPB74****^3^*** | 73.00 | 68.4 |
| *S. clavuligerus* ATCC27064 | 71.94 | 67.7 |
| *S. pristinaespiralis* ATCC25486 | 71.16 | 66.4 |
| *S. sviceus* ATCC29083 | 70.46 | 64.9 |
| *S. ghanaensis* ATCC14672 | 72.22 | 66.7 |
| *S. roseosporus* NRRL15998 | 71.36 | 66.7 (SSGG_02178) |
| *S. roseosporus* NRRL15998***^3^*** |  | 67.8 (SSGG_05025) |
| *S. albus J1074* | 73.15 | 68.3 (SSHG_04184) |
| *S. albus J1074* |  | 68.8 (SSHG_04506) |
| *S. sp. SPB78* | 72.90 | 68.2 |
| *S. griseoflavus* Tu4000 | 71.67 | 66.8 |
| *S. lividans* TK24 | 72.18 | 66.8 |
| *S. viridochromogenes* DSM40736***^3^*** | 71.12 | 66.1 |
| *S. sp.* E14 | 72.67 | 69.3 |
| *S. griseus* NBRC 13350 | 72.2 | 67.4 (SGR_4703) |
| *S. griseus* NBRC 13350 |  | 68.4 (SGR_2126) |
| *S. avermitilis* | 70.7 | 67.7 |
| *S. venezuelae* ATCC10712 | 72.4 | 68.3 |
| *S. bingchenggensis* | 70.8 | 67.4 |
| *S. cattleya* | 72.9 | 72.6 |
| *S. scabie* | 71.5 | 67.6 |
| *S. hygroscopicus****^2^*** | 71.9 | 65.1 |
| *S. violaceusniger****^1^*** | 71 | 65.1 |
| *S. sp* Tu6071 | 73.1 | 68.4 |
| *Stigmatella aurantiaca****^2^*** | 67.5 | 62 |

1. No cognate *cslA*
2. No cognate endoglucanase
3. Endoglucanse gene not adjacent to *glxA*
